# Influence of pH and lysis duration on DNA damage detection: a comparison of neutral and alkaline comet assays

**DOI:** 10.1093/mutage/geaf019

**Published:** 2025-08-22

**Authors:** Ruzica Pribakovic, Julia Bornhorst, Helga Stopper, Ezgi Eyluel Bankoglu

**Affiliations:** Institute of Pharmacology and Toxicology, University of Wuerzburg, 97078 Wuerzburg, Germany; Food Chemistry with Focus on Toxicology, Faculty of Mathematics and Natural Sciences, University of Wuppertal, 42119 Wuppertal, Germany; Food Chemistry with Focus on Toxicology, Faculty of Mathematics and Natural Sciences, University of Wuppertal, 42119 Wuppertal, Germany; Institute of Pharmacology and Toxicology, University of Wuerzburg, 97078 Wuerzburg, Germany; Institute of Pharmacology and Toxicology, University of Wuerzburg, 97078 Wuerzburg, Germany; Unit Safety of Food Contact Materials, Department Food and Feed Safety in the Food Chain, German Federal Institute for Risk Assessment, 10589 Berlin, Germany

**Keywords:** comet assay, DNA damage, alkaline unwinding, neutral comet assay, alkaline comet assay

## Abstract

The comet assay is a widely used method for measuring DNA damage and DNA repair. When DNA strand breaks happen, the supercoiling of DNA is relaxed, and after alkaline or neutral electrophoresis, depending on the type of performed comet assay, DNA moves toward the anode, forming a comet tail. Thus, with increasing frequency of DNA strand breaks, an increase in the percentage of DNA in the tail is observed. The aim of this study was to compare systematically various steps like lysis, duration of electrophoresis, and pH of the electrophoresis solution and their effect on the comet tail with regard to sensitivity for detection and quantification of DNA damage. We treated human lymphoblastoid TK6 cells with known genotoxic substances with a different mode of action and then performed both standard and modified alkaline and neutral comet assays. The modifications included *Fpg-* and *MspI*-modified comet assays. Several aspects of this comparison are investigated for the first time here. The results obtained from these experiments showed a higher %DNA in tail in the alkaline comet assay compared to the neutral comet assay. Additionally, the lysis step was not critical in the alkaline comet assay, whereas it was essential for the neutral comet assay. Results from alkaline *Fpg*-modified comet assay showed higher sensitivity in detecting single strand breaks and the neutral *MspI*-modified comet assay was better in detecting DNA double-strand breaks. Overall, our findings provided valuable insight into the differences between alkaline and neutral electrophoresis conditions in the comet assay and indicated that the alkaline comet assay is more sensitive for measuring total DNA damage.

## Introduction

The comet assay, also known as single-cell gel electrophoresis, is a sensitive, simple, and cost-effective tool for analyzing and quantifying DNA damage and it can be applied to almost all eukaryotic cells and tissues, as long as a single cell suspension can be obtained. The assay is widely used in genotoxicity, in both *in vitro* and *in vivo* studies and human biomonitoring, also for investigating mechanisms of DNA damage and DNA repair [1, 2].

Following the exposure to test substances and DNA damaging agents, the assay starts with the preparation of microscope slides where cells are embedded in an agarose gel. Cells are then lysed in a cold lysis solution in order to remove membranes and to allow DNA with relaxed supercoiling to migrate out of the nucleus. In alkaline comet assay, an unwinding step is carried out to convert the DNA into a single stranded form. The more breaks are present, the more DNA migrates out of the nucleus. With electrophoresis applied, DNA is then drawn toward the anode, forming an electrophoretic track, which after staining with a suitable dye, is visible under the fluorescence microscope as a comet tail. The sum of the fluorescence intensity in the tail region compared to that of the whole comet represents the amount of DNA damage. The comet assay detects a variety of DNA lesions, from DNA single and double-strand breaks, oxidative lesions to alkali-labile sites [3]. Depending on the pH of the electrophoresis step, there are two main forms of comet assay: neutral, where the electrophoresis is performed at a neutral pH (7.4 [4], 7.5 [5, 6], 8 [7], or 8.4 [8]), and alkaline with electrophoresis under alkaline conditions, usually at pH >13 [9]. A widely discussed issue is the type of DNA strand break that can be detected in the comet assay depending on the pH of the electrophoresis step. The most common option is that by conducting the neutral comet assay, only double-strand DNA breaks are detected, while the alkaline comet assay can determine single and double DNA strand breaks, plus alkali-labile sites which are also being converted into single strand breaks.

The comet assay can also be modified to detect and measure a wider range of DNA lesions. This is achieved with lesion-specific enzymes, such as formamidopyrimidine DNA glycosylase (*Fpg*), which detects lesions referred as *Fpg*-sensitive sites (8-oxo-7,8-dihydroguanine and certain types of formamidopyrimidine nucleobases), yielding a DNA single strand break, and it is commonly used to identify oxidative DNA damage [10]. The lesion-specific endonuclease is *MspI*, a methyl-sensitive restriction endonuclease, that digests the 5′-CmCGG-3′ sequence and induces DNA strand breaks on both DNA strands. It is usually used for the analysis of the global DNA methylation level [11]. However, here it is applied to induce specifically double-strand breaks on the DNA.

We investigated how different experimental conditions including lysis, pH of electrophoresis solution, and electrophoresis duration influence the comet tail. For this aim, we used the human lymphoblastoid cell line TK6 and conducted comet assay after treating with selected reference genotoxins with different mode of actions under both alkaline and neutral conditions. Using the enzymes *Fpg* and *MspI*, we inserted specific single and double DNA strand breaks. Finding of these study presents valuable information on influence of experimental conditions on the comet tail and compares the neutral and alkaline electrophoresis for their sensitivity. To our knowledge, this is the first study to directly compare alkaline and neutral comet assay conditions within a unified experimental setting using well-characterized reference genotoxins with different mode of actions, thereby providing novel insights into the assay and its applicability in genotoxicity testing.

## Material and methods

### Materials

Dimethyl sulfoxide (DMSO), normal melting point agarose, sodium hydroxide and fully frosted slides were obtained from Carl Roth (Karlsruhe, Germany). GelGreen Nucleic Acid Stain and GelRed Nucleic Acid stain were purchased from Biotium (Darmstadt, Germany). Fetal calf serum was obtained from Anprotec (Bruckberg, Germany). Formamidopyrimidine DNA glycosylase (*Fpg*) was purchased from New England Biolabs (Frankfurt, Germany) and *MspI* enzyme was purchased from ThermoFisher (Frankfurt, Germany). All other substances were obtained by Sigma-Aldrich (Steinheim, Germany), unless indicated otherwise.

### Methods

#### Cell culture

Human lymphoblastoid cell line TK6 was cultured in RPMI medium supplemented with 10% (v/v) fetal calf serum, 1% (w/v) l-glutamine, 1% (w/v) sodium pyruvate, and 0.4% (w/v) penicillin and streptomycin, and kept in the humidified incubator with 5% CO_2_ at 37°C. Experiments were conducted using TK6 cells with a passage number up to 30. One day before the experiment, 200 000 cells were seeded in a six-well plate and next day, cells were treated with the selected test substances [4 h with methyl methane sulfonate (MMS: 100 μM), 4 h with etoposide (1 μM), 4 h with staurosporine (1 μM), 1 h with *tert*-butyl hydroperoxide (tBOOH: 25 μM), 1 h with bleomycin (0.5 μg/ml) and 1 h with potassium-bromate (0.5 mM)].

#### Viability test

Cell viability was done at the time of cell harvest for comet assay. After collecting cell suspension for comet assay, 35 μl of cell suspension was mixed with 15 μl of staining solution [2 μl GelRed nucleic acid stain (10 000× in water) and 12 μl fluorescein diacetate (5 mg/ml in acetone)] and then 15 μl of this mixture was immediately used for preparing microscope slide for scoring. In total, 200 cells per slide from each replicate were scored by using an Eclipse 55i microscope (Nikon GmbH, Dusseldorf, Germany) at 200-fold magnification with a FITC filter. The percentage of vital cells was determined by counting red and green stained cells.

#### Comet assay

The protocol for comet assay was previously published Bankoglu *et al*. [12]. After treatment, cells were washed with cold phosphate buffered saline (PBS), number of cells was determined as described above for each sample and adjusted to ensure even cell distribution for easier evaluation afterward. For the preparation of minigels, 50 μl of cell suspension was mixed with 120 μl of pre-warmed low melting point agarose (1%) and then 5 μl of the mixture was pipetted for forming minigel on a Superfrost slide, previously coated with 1.5% normal melting point agarose. To prepare single gels (only for *MspI* comet assay), 20 μl of cell suspension was mixed with 180 μl of pre-warmed low melting point agarose (0.8%) and then 45 μl of that mixture was used for preparing comet slides. Slides were incubated for 1 h in a lysis solution (1% Triton X-100, 10% dimethyl sulfoxide and 89% lysis buffer: 10 mM Tris, 2.5 M NaCl and 100 mM Na_2_EDTA, pH 10). After lysis, half of the slides underwent alkaline electrophoresis and the other half neutral electrophoresis. All experimental conditions tested throughout this study, including treatment types, concentrations, and variations in lysis and electrophoresis duration under both alkaline and neutral comet assay conditions are summarized in Table 1.

**Table 1:** Overview of experimental conditions tested for each comet assay type.

**Comparison**	**Assay type**	**Treatments**	**Lysis**	**Electrophoresis**
**Different substances**	Alkaline comet assay and neutral comet assay (minigels)	100 μM MMS for 4 h	1 h at 4°C	30 min on ice
		1 μM Etoposide for 4 h		
		25 μM tBOOH for 1 h		
		0.5 μg/ml Bleomycin for 1 h		
		1 μM Staurosporine for 1 h		
**Electrophoresis duration**	Alkaline comet assay and neutral comet assay (minigels)	100 μM MMS for 4 h	1 h at 4°C	0, 10, 20, 30, 40, 60 min on ice
		1 μM Staurosporine for 1 h		
**Lysis duration**	Alkaline comet assay and neutral comet assay (minigels)	100 μM MMS for 4 h	0, 5, 10, 20, 40, 60 min at 4°C	30 min on ice
		25 μM tBOOH for 1 h		
		0.5 μg/ml Bleomycin for 1 h		
**DNA single strand breaks**	Alkaline *Fpg* comet assay and neutral *Fpg* comet assay (minigels)	0.5 mM KBrO₃ for 1 h	1 h at 4°C	0, 10, 20, 30, 40, 60 min on ice
**DNA double-strand breaks**	Alkaline *Msp-I* comet assay and neutral *Msp-I* comet assay (single gel format)	No substance treatment Incubating either with 100 μl of 1.5 U MspI enzyme or with 100 μl of Tango buffer for 1 h at 37°C	1 h at 4°C	0, 10, 20, 30, 40, 60 min on ice

##### Alkaline comet assay

After lysis step, slides were placed in a horizontal electrophoresis chamber filled with cold alkaline (1 mM Na_2_EDTA, 300 mM NaOH, pH > 13) electrophoresis solution and incubated for 20 min for alkaline unwinding and then electrophoresis was conducted (1 V/cm, 20 min). To evaluate the effect of lysis step and electrophoresis duration, different time points were tested: 0, 5, 10, 20, 40, and 60 min for lysis and 0, 10, 20, 30, 40, and 60 min for electrophoresis.

The slides with single gel were neutralized in PBS for 5 min and then dehydrated in ice cold methanol for 5 min. The slides with minigels were washed in PBS, then in bidistilled water each for 10 min. For dehydration of minigels, slides were placed in 70% ethanol for 15 min, then in 100% ethanol for 30 min. All slides (single gel and minigel) were stained with GelRed nucleic acid stain solution after air-drying. The staining solution was prepared by diluting GelRed nucleic acid stain (10 000× in water) at a ratio 1:100 and then mixing with diazabicyclo octane at a ratio of 1:4. The percentage of DNA in tail was scored by using Komet 6 software (BFI Optilas, Germany) in 50 random nuclei (25 per replicate) per sample.

##### Neutral comet assay

In case of neutral comet assay, after lysis, slides were washed three times each for 5 min, with neutral electrophoresis solution (90 mM TRIS, 90 mM boric acid, 2 mM Na_2_EDTA, pH 7.5) and then electrophoresis was performed for 20 min (1 V/cm) in neutral conditions. To evaluate the effect of lysis step and electrophoresis duration, different time points were tested: 0, 5, 10, 20, 40, and 60 min for lysis and 0, 10, 20, 30, 40, and 60 min for electrophoresis.

After electrophoresis, in both assays, slides were incubated for 10 min in cold PBS, then 10 min in cold distilled water, then 15 min in cold 70% v/v ethanol and 30 min in cold absolute ethanol, for dehydrating the mini-gels before staining. Slides were then stained with GelRed for scoring. The staining solution was prepared by diluting GelRed nucleic acid stain (10 000× in water) at a ratio 1:100 and then mixing with diazabicyclo octane at a ratio of 1:4. For scoring we used Komet 6 software and 50 nuclei per sample were scored (25 nuclei per each replicate) and the results were presented as %DNA in tail.

##### *Fpg* comet assay

For the *Fpg*-modified comet assay, TK6 cells were treated with 0.5 mM potassium bromate for 1 h. After treatment, the cells were collected and microscope slides were prepared. All subsequent steps up to electrophoresis were carried out as described above. After the lysis step, slides were incubated in a Coplin jar either with enzyme reaction buffer (40 mM HEPES, 0.1 M KCl, 0.5 mM EDTA, 0.2 mg/ml BSA, pH 8.0) or with *Fpg* enzyme (100 ml; 1:30 000 dilution from the original stock with a concentration of 8000 IU/ml) for 1 h, at 37°C. Both neutral and alkaline comet assays were performed as explained above, but with a different durations of electrophoresis (0, 10, 20, 30, 40, and 60 min). The scoring was performed as described above and the results were presented as %DNA in tail.

##### *MspI* comet assay

For *MspI* comet assay, microscope slides were prepared as described above only by using solvent control group. Slides were then put in a cold lysis solution for 1 h, at 4°C. After the lysis step, either 100 μl of 1.5 U *MspI* enzyme or 100 μl of Tango Buffer was applied to each slide and covered with a glass coverslip, then slides were left in a humid box at 37°C for 1 hour. Both alkaline and neutral comet assays were conducted with a duration of electrophoresis of 0, 10, 20, 30, 40, and 60 min. This experiment was repeated for three times. One time the same experiment was repeated with a longer electrophoresis duration: 0, 30, 60, 80, 100, and 120 min. The scoring was performed as above and the results were presented as %DNA in tail.

#### Data analysis and statistics

Graphics were drawn using software GraphPad Prism (version 9.5.1). At least three independent experiments were performed in each case and results were represented as Mean ± SD. Statistical analysis was performed with GraphPad Prism (version 9.5.1) under the assumption of normal distribution. One-way ANOVA was used to compare the selected substances in the alkaline and neutral comet assay. Two-way ANOVA followed by Tukey’s multiple comparison test was applied to assess the effects of electrophoresis conditions and lysis durations on DNA damage in both alkaline and neutral comet assays as well as to compare the alkaline and neutral *MspI-* and *Fpg-*modified comet assays under varying electrophoresis durations. A *P*-value of ≤.05 was considered statistically significant.

## Results

### Comparing selected substances and induced DNA damage in alkaline and neutral comet assay

We used well-known reference compounds to induce various DNA lesions and observe sensitivity differences in alkaline and in neutral electrophoresis in the comet assay. For this aim, MMS was used as alkylating agent [13], etoposide as topoisomerase II inhibitor [14, 15] which can induce DNA double-strand breaks (DSBs) in addition to a majority of single strand breaks, bleomycin as a cytostatic medication [16, 17], which can induce both single and double-strand breaks and tBOOH was used as an oxidizing agent [18].

**Figure 1:**
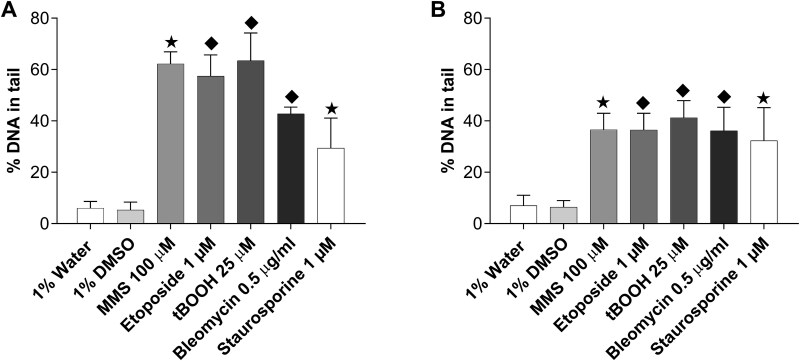
Comparing selected substances in (A) alkaline and (B) neutral comet assay in TK6 cell line. 50 nuclei are scored per sample with results presented as %DNA in tail. Mean ± SD values obtained from three independent experiments are represented. Statistical significance is depicted as ♦*P* ≤ .05 vs. 1% water. ★*P* ≤ .05 vs. 1% DMSO.

TK6 cell line was treated with 100 μM MMS for 4 h and 1 μM etoposide, 25 μM tBOOH, 0.5 μM bleomycin, and 1 μM staurosporine for 1 h. The treatment concentrations and durations have been selected according to the previous work and experience within the working group [12]. Only staurosporine treatment caused a significant rise in the percentage of dead cells, reaching 11.25% (Table 2), while other treatments did not cause elevated cell death. After collecting cells, samples were prepared both for alkaline and neutral comet assay for direct comparison. The percentage of DNA in tail can be seen in Fig. 1. All selected compounds induced a significant increase in DNA damage compared to their solvent control in the alkaline as well as in the neutral comet assay. However, the percentage of DNA in tail was slightly higher in the alkaline comet assay compared to the neutral one, except for staurosporine. The neutral comet assay showed very similar results for all selected compounds independent from their mode of action.

**Table 2:** Percent of dead cells after treatment with selected substances.

**Samples**	**Percentage of dead cells (%)**
**1% Water**	3.25 ± 0.66
**1% DMSO**	4.94 ± 1.25
**MMS 100 μM**	4.50 ± 0.66
**Etoposide 1 μM**	6.08 ± 0.14
**tBOOH 25 μM**	5.92 ± 1.81
**Bleomycin**	3.84 ± 0.88
**Staurosporine 1 μM**	11.25 ± 2.59^*^

^*^ *P* ≤ .05 vs. 1% DMSO.

### Effects of lysis and electrophoresis duration in alkaline and neutral comet assay

**Figure 2:**
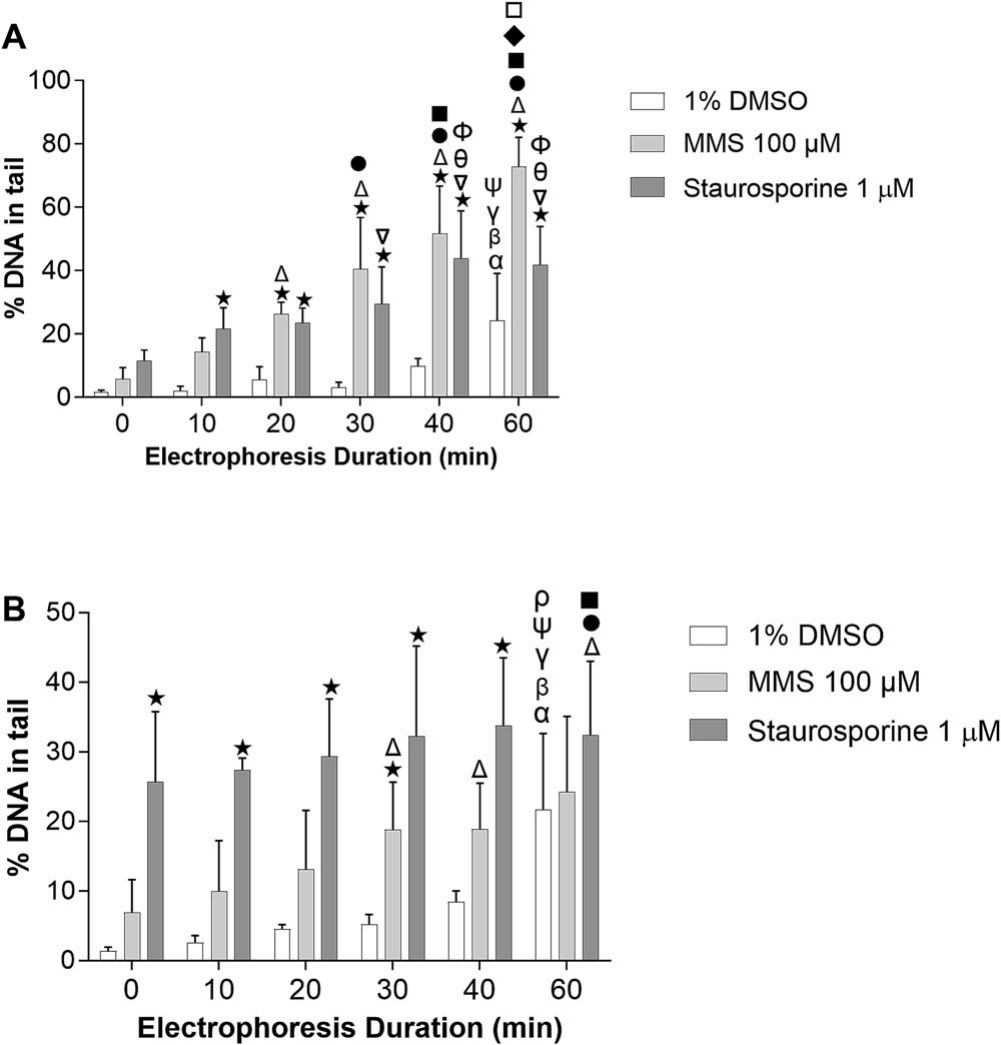
Effects of the different electrophoresis duration on the DNA damage detected by (A) alkaline and (B) neutral comet assay on TK6 cell line. Cells were treated with 100 μM MMS and 1 μM staurosporine for 4 h and electrophoresis was conducted up to 60 min. Results are presented as %DNA in tail and the values represent the Mean ± SD of four experiments. Statistical significance is depicted as ★*P* ≤ .05 vs. 1% DMSO (at the same time point), α*P* ≤ .05 vs. 0 min (1% DMSO), β*P* ≤ .05 vs. 10 min (1% DMSO), γ*P* ≤ .05 vs. 20 min (1% DMSO), Ψ*P* ≤ .05 vs. 30 min (1% DMSO), ρ*P* ≤ .05 vs. 40 min (1% DMSO), Δ*P* ≤ .05 vs. 0 min (MMS), $ \bullet$*P* ≤ .05 vs. 10 min (MMS), ■*P* ≤ .05 vs. 20 min (MMS), ♦*P* ≤ .05 vs. 30 min (MMS), □*P* ≤ .05 vs. 40 min (MMS), ∇*P* ≤ .05 vs. 0 min (Staurosporine), θ*P* ≤ .05 vs. 10 min (Staurosporine) and Φ*P* ≤ .05 vs. 20 min (Staurosporine).

**Figure 3:**
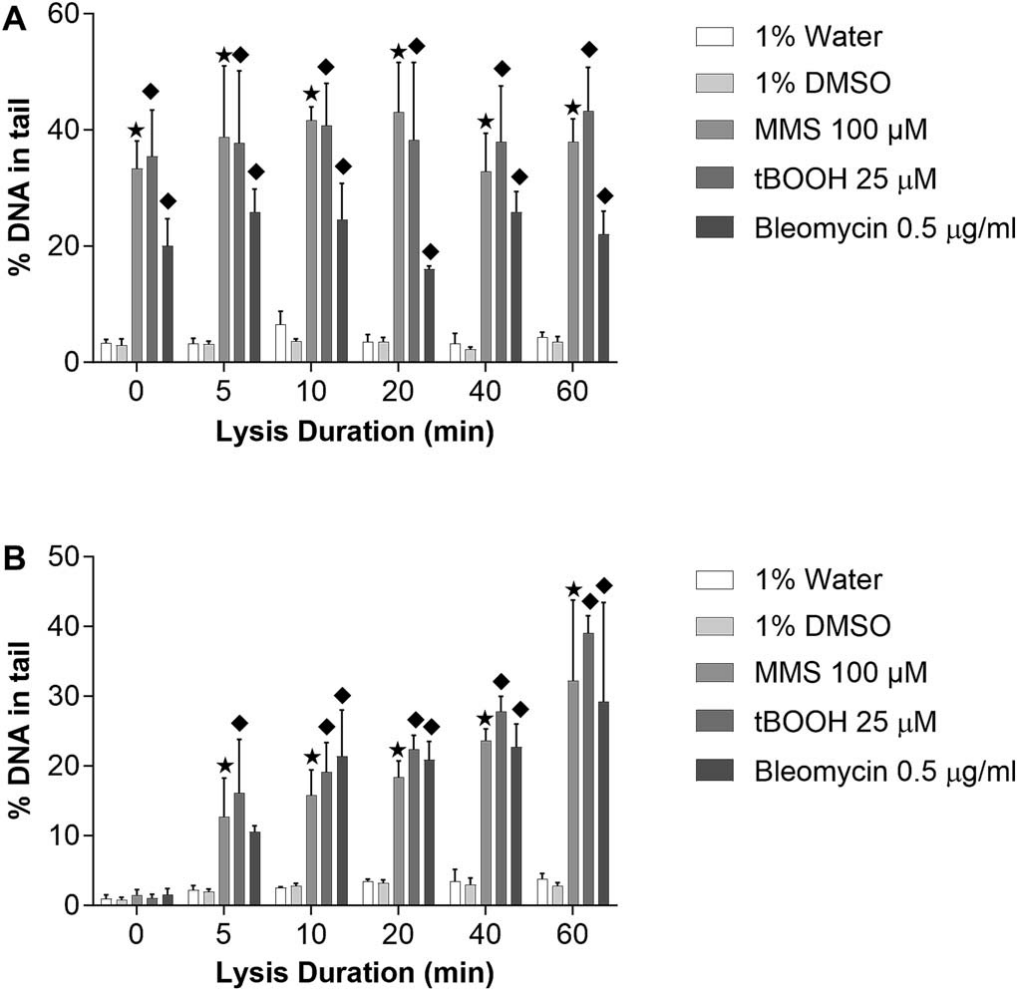
Effect of the different lysis durations, from 0 to 60 min of lysis, after treating TK6 cells with 100 μM MMS, 25 μM tBOOH and 0.5 μg/ml bleomycin in (A) alkaline and (B) neutral comet assay. Mean ± SD values obtained from three independent experiments are represented. Statistical significance is depicted as ◆*P* ≤ .05 vs. 1% water. ★*P* ≤ .05 vs. 1% DMSO.

To investigate the sensitivity differences between the alkaline and the neutral comet assay, we studied the effects of varying electrophoresis (Fig. 2) and lysis durations (Fig. 3). We focused on MMS as an alkylating agent and a clastogen to induce DNA damage and staurosporine as non-genotoxic apoptosis inducer to induce DNA fragmentation for investigating the effect of electrophoresis durations. The electrophoresis was conducted under both alkaline and neutral conditions for 0, 10, 20, 30, 40, and 60 min (Fig. 2). Our results showed a slight increase in the percentage of DNA in tail with longer electrophoresis durations. After 60 min of electrophoresis, there was an increase in the percentage of DNA in tail in the solvent controls for both assays. Both substances caused gradually increased % DNA in tail with longer electrophoresis durations under alkaline condition (Fig. 2A). This was not the case in the neutral version, where only MMS caused a time dependent further increase (Fig. 2B). Staurosporine treated samples showed significantly higher percentage of DNA in tail, even without electrophoresis in both conditions, but with about the same intensity at all electrophoresis durations in the neutral version, suggesting the presence of diffusing fragments in the agarose.

In comparison between alkaline and neutral comet assay, MMS yielded higher damage in the alkaline, while the damage caused by staurosporine was similar under both conditions, however not dependent on electrophoresis duration.

For a better understanding of how crucial the lysis step is in the comet assay, we investigated the effect of different lysis durations on comet tail, in both alkaline and neutral comet assay. After treating TK6 cells with 100 μM MMS for 4 h, 25 μM tBOOH and 0.5 μM bleomycin for an hour, microscope slides were prepared for lysis durations of 0, 5, 10, 20, 40, and 60 min. After the lysis step, both assays were conducted in independent experiments with the electrophoresis duration of 30 min (Fig. 3). The lysis duration had a small effect on the percentage of DNA in tail for the solvent controls in both alkaline and neutral electrophoresis.

Results obtained from alkaline comet assay, as presented in the Fig. 3A showed significant increase of %DNA in tail in all time points of lysis duration. Similar %DNA in tail was observed in all treatments, even in samples without lysis (0 min). Results obtained in the neutral comet assay (Fig. 3B) showed that in samples without lysis, there was almost no comet tail detectable, while from 5 to 60 min of lysis, it increased gradually in all treatments.

### *Fpg-* and *MspI*-modified comet assays

To understand the influence of the presence of single- and double-strand breaks in the context of sensitivity differences between the alkaline and neutral comet assay, we conducted the *Fpg*-modified comet assay as well as *MspI*-modified comet assay under alkaline and neutral conditions. For the *Fpg*-modified comet assay, TK6 cells were treated with 0.5 mM potassium bromate for an hour to induce oxidative DNA lesions, which would be recognized and cut by DNA glycosylase *Fpg* due to its base excision activity. By this way, we could induce mostly single DNA strand breaks. *MspI* is a bacterial isoschimeric restriction endonuclease that recognizes 5′-CCGG-3′ tetranucleotide sequence. It digests 5′-CmCGG-3′ but not 5′mCCGG-3′ [8], and can cleave DNA at the internal C residue of its recognition sequence CCGG on both opposite DNA strands and by this way induces DSBs. Both modified assays were conducted under neutral and alkaline conditions with different durations of electrophoresis (0, 10, 20, 30, 40, and 60 min).

**Figure 4:**
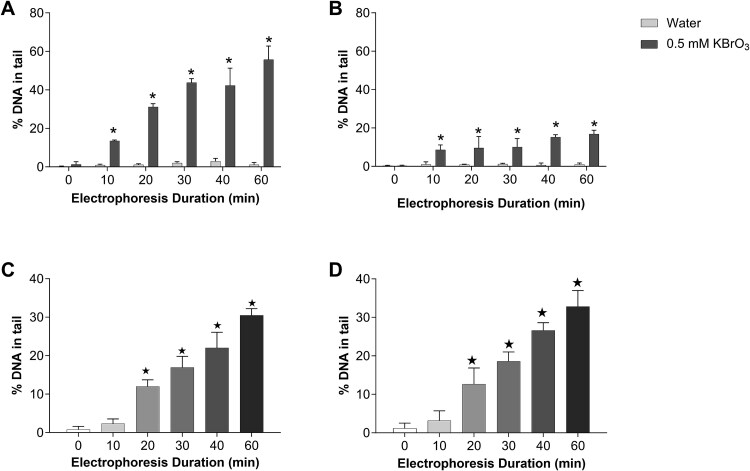
(A) Alkaline and (B) neutral Fpg modified comet assay with different duration of electrophoresis. TK6 cells were treated with 0.5 mM potassium bromate, then either alkaline or neutral Fpg comet assay was conducted with electrophoresis durations from 0 to 60 min. Mean ± SD values obtained from three independent experiments are represented. ★*P* ≤ .05 vs its control. (C) Alkaline and (D) neutral *Mspi* modified comet assay with different duration of electrophoresis. TK cells were incubated with Mspi endonuclease, then either alkaline or neutral *MspI* comet assay was conducted from 0 to 60 min. Mean ± SD values obtained from three independent experiments are represented. Statistical significance is depicted as ★*P* ≤ .05 vs 0 min.

The results obtained in the *Fpg* alkaline comet assay in a combination with a different electrophoresis durations are shown in Fig. 4A and B. Both revealed a gradual increase in DNA damage with increasing electrophoresis duration, with a much smaller extent of DNA damage in the neutral version. Thus, alkaline conditions led to an increased sensitivity for detecting single strand breaks compared to neutral.

In order to compare sensitivity to the detection and measurement of double-strand DNA breaks, we conducted alkaline and neutral *MspI* comet assays in combination with different electrophoresis durations. The percentage of DNA in tail gradually increased with increasing electrophoresis durations under both alkaline and neutral conditions (Fig. 4C and D). There was no significant difference between neutral and alkaline conditions in this endpoint. This leads to the conclusion that both alkaline and neutral electrophoresis can detect DSBs.

## Discussion

The comet assay is a widely used tool for measuring the DNA damage and the two main types of comet assay are alkaline and neutral comet assay. The neutral comet assay has been often considered to be specific for the DSBs, while the alkaline comet assay enables the detection of DNA single and double-strand breaks, together with alkali-labile sites. We put some effort to understand the effect of pH and duration of electrophoresis as well as lysis steps on the outcome of the comet assay and the type of the detected damage. For this, we conducted and compared alkaline and neutral comet assays, after treating TK6 cells with selected well known reference substances with different mode of actions. Furthermore, we applied enzyme modifications of the assay to gain further insight into how different forms of DNA damage, such as single or double-strand breaks, contribute to the formation of DNA in the comet tail under alkaline or neutral electrophoresis conditions.

As first, we investigated the effect of different electrophoresis solutions on the comet tail. The alkaline comet assay showed greater sensitivity to DNA damage, by giving a higher %DNA in tail for all treatments. This might also be explained by the idea that some of the lesions like alkali-labile sites became only visible under alkaline conditions. In addition, during the alkaline comet assay, DNA molecules are single stranded due to the alkaline unwinding and this can lead to easier movement during the electrophoresis compared to the double-stranded DNA molecule in the neutral comet assay. Therefore, the alkaline comet assay can exhibit a higher percentage of DNA in the comet tail compared to the neutral comet assay caused by the same amount of DNA damage [19].

Staurosporine is a potent protein kinase inhibitor, which can induce apoptosis leading to DNA fragmentation. This makes it a useful test substance for studying DNA fragments under both alkaline and neutral conditions. Our hypothesis was that the comet tail formation might reach a saturation point for the MMS-treated cells compared to staurosporine-treated cells during longer electrophoresis, due to the length of the DNA loops in the case of MMS, while staurosporine might induce freely moving (apoptotic) DNA fragments. However, our results are not in line with this expectation. Staurosporine treatment yielded so called ghost cells which bear a small head and a huge broad tail coming from heavily damaged cells or apoptotic cells [20]. The longer electrophoresis durations increased the background DNA damage in control cells, complicating the analysis and limiting the duration of the electrophoresis. Although we had several limitations, we had some useful observations with the staurosporine treated cells. In the alkaline comet assay, staurosporine induced DNA fragments may be smaller than in the neutral comet due to unwinding into single stranded DNA. This could result in more ghost cells and unintentional selection of cells with lower damage for scoring. In contrast, DNA fragments in the neutral comet assay may be more stable and visible due to their double-stranded structure. This may explain the significant increase in the %DNA in tail for staurosporine treated cells without electrophoresis in neutral conditions. Furthermore, in the neutral comet assay, the percentage of DNA in the comet tail plateaued after 30 min of electrophoresis. In the alkaline comet assay, the percentage of DNA in the comet tail continued to increase with longer electrophoresis durations.

As next, we investigated the effect of the different lysis duration on the comet tail. Results obtained from alkaline comet assay showed significant increase of %DNA in tail in all time points of lysis duration. Similar %DNA in tail was observed in all treatments, even in samples without lysis, which leads to the conclusion that lysis step is not crucial in the alkaline comet assay. Azqueta *et al*. [21] studied unwinding time and electrophoresis conditions to optimize the alkaline comet assay. Their findings showed that although the 40 min alkaline unwinding time is recommended, shorter duration can be used. Enciso *et al*. [22] studied the effect of different times of lysis on % DNA in the tail by the comet assay in HeLa cells after MMS and hydrogen peroxide treatments. Their findings were in parallel with our observation that performing alkaline comet assay without lysis step can give reliable results. In contrast to the alkaline comet assay, results obtained with the neutral comet assay showed that in samples without lysis, there was no measurable comet tail, while from 5 to 60 min of lysis, it was increased gradually in all treatments. With rising sensitivity to DNA damage with a longer duration of lysis, we concluded that the lysis step is essential in the neutral comet assay. The need for lysis in the neutral comet assay may indicate that the comet tail under neutral conditions consists of DNA loops that are attached to the nuclear matrix.

Finally, we conducted *Fpg-* and *MspI*-modified comet assays, both alkaline and neutral, to understand the sensitivity differences in the detection of single and double-strand breaks. In order to induce mostly single strand breaks, we used potassium bromate treated cells. By this way, we aimed to generate nucleobase oxidation, which are then transformed into single strand breaks after the incubation with *Fpg* due to its base excision repair activity. This allowed us to compare the alkaline and the neutral comet assays in sensitivity to detect single DNA strand breaks. Results of both assays showed gradually increased %DNA in tail with a longer electrophoresis durations, but in the case of the alkaline comet assay, comet tails were longer in all electrophoresis time points. This means that the alkaline Fpg-modified comet assay was more sensitive in detecting and measuring single strand breaks. We incubated cells with *MspI*, a bacterial restriction enzyme, which digests 5′-CmCGG-3′ sequence, causing mostly double-strand breaks. This allowed us to compare alkaline and neutral comet assays in the term of sensitivity toward double-strand DNA breaks. Results obtained in both alkaline and neutral comet assay showed gradually increased %DNA in tail with a longer electrophoresis durations. The neutral comet assay yielded a slightly higher comet values in all measured electrophoresis time points, however there was no significant difference between neutral and alkaline conditions. This leads to the conclusion that both alkaline and neutral comet assay can detect DSBs, whereas the alkaline comet assay is more sensitive for detecting other lesions such as alkali-labile sites.

In conclusion, our results showed that the alkaline comet assay had greater sensitivity for the detection of DNA damage compared to the neutral comet assay. This increased sensitivity under alkaline condition highlights the assay’s ability to detect a broader spectrum of DNA lesions, making the alkaline comet assay suitable for genotoxicity assessment in various context. Moreover, by applying enzyme modifications, we gained additional insights into how specific types of DNA damage contribute to DNA migration during electrophoresis under different assay conditions. These observations support the relevance of electrophoresis step particularly pH in effecting the assay’s performance. Overall, our study provides a systematic comparison between alkaline and neutral comet assays and advanced methodological understanding of the comet assay, offering practical recommendations for improving its application in DNA damage detection.
